# The synergistic effects of *Guaiacum officinale* and *Rhodomyrtus tomentosa* extracts in the treatment of acne vulgaris on sensitive skin

**DOI:** 10.1111/jocd.16394

**Published:** 2024-05-24

**Authors:** Jianhua Zhang, Shichao Liu, Wenjiao Guo, Na Li

**Affiliations:** ^1^ N.O.D topia (GuangZhou) Biotechnology Co., Ltd. Guangzhou China; ^2^ Simpcare (GuangZhou) Biotechnology Co., Ltd. Guangzhou China

**Keywords:** acne vulgaris, anti‐inflammation, *Guaiacum officinale*, *Rhodomyrtus tomentosa*, sensitive skin

## Abstract

**Background:**

Acne vulgaris, a common chronic dermatological condition worldwide, is associated with inflammatory response and *Cutibacterium acnes*. Individuals with acne vulgaris and sensitive skin have limited suitable treatments due to the skin irritation and side effects exhibited by current hydroxy acidic medications.

**Aims:**

This study aimed to evaluate the synergistic effects of *Guaiacum officinale* (GO) and *Rhodomyrtus Tomentosa* (RT) extracts for treating acne vulgaris on sensitive skin by inhibiting inflammation.

**Methods:**

The phytochemical constituents and antioxidant activity of GO and RT extracts were determined in vitro. The anti‐inflammatory effects were investigated in peptidoglycan (PGN)‐induced HaCaT cells. Further, a 28‐day clinical trial was conducted involving 30 subjects with both sensitive skin and acne to evaluate the efficacy and subjects' satisfaction.

**Results:**

Total phenolics and flavonoids were detected in GO and RT extracts, the IC_50_ values for DPPH radical scavenging were 6.15 wt% and 0.76 wt%, respectively. The combination of GO and RT extracts at a 1:1 (v/v) ratio significantly decreased the expression of *TLR‐2* and *TLR‐4*, as well as the secretion of IL‐1α, IL‐8, and TNF‐α in PGN‐induced HaCaT cells, by 2.30–7.93 times compared to GO extract alone (*p* < 0.05). Moreover, the cream containing 5 wt% the combination significantly improved facial acne and redness (*p* < 0.05). The number of comedones decreased by 50.00% and papules by 30.65% after 28 days of application. No adverse events were reported and 96.67% of the subjects were satisfied with the treatment.

**Conclusion:**

The efficacy of the GO and RT extracts in synergistically suppressing inflammation, improving acne vulgaris, and reducing redness. The study offers an effective and non‐irritant treatment for acne vulgaris in individuals with sensitive skin.

## 1 INTRODUCTION

Acne vulgar, a chronic inflammatory disease of the pilosebaceous unit, characterized by the formation of open and closed comedones, papules, pustules, nodules, and cysts. It is among the most common dermatological conditions worldwide. It's reported to afflict about 80% of adolescents and young adults. In recent years, the prevalence of acne has been increasing in the adult population, especially in the female population, with rates as high as 40%–50%.^1^ Acne vulgaris primarily occurs on the face, neck, arms, upper chest, and back, leading to scarring and post‐inflammatory hyperpigmentation. Facial acne not only impacts physical appearance but also presents symptoms such as itching, erythema, and pain, affecting overall health and quality of life.^2^

The complex pathogenesis of acne vulgaris remains incompletely understood, but research suggests that inflammation and *Cutibacterium acnes* (*C. acnes*, formerly *Propionibacterium acnes*) play pivotal roles in its development. Dysregulation of the *C. acnes* colonization environment within hair follicles may be a major factor in the formation of acne vulgaris and hinder the process of wound healing in the skin.^3^ *C. acnes*, a gram‐positive anaerobic bacterium, exerts a substantial impact on the inflammatory phase of acne. Peptidoglycan (PGN), a key component of the *C. acnes* cell wall, activates Toll‐like receptors 2 and 4 (*TLR‐2* and *TLR‐4*), thereby triggering the MAPK and Nuclear Factor κB (*NF‐κB*) pathways.^4^ This activation leads to the production of proinflammatory cytokines, including IL‐1, IL‐6, IL‐8, TNF‐α, and HBD‐2, initiating chemotactic activity in inflammatory cells.^5^

Recent study indicates a high incidence rate of acne in sensitive skin.^6^ For those affected by acne vulgaris, the skin barrier could be compromised by frequent acne outbreaks, which could reduce immune defense capacity and heighten sensitivity. This could lead to persistent inflammation or recurrent acne, creating a vicious cycle of acne vulgaris and sensitive skin. Conventional potent medications such as hydroxy acidic acid (salicylic acid, fruit acids, and azelaic acid), isotretinoin, benzoyl peroxide, chemical exfoliants, and laser treatments for acne treatment may induce skin irritation and side effects,^7^ presenting challenges for individuals with both acne‐prone and sensitive skin. Therefore, as highlighted by Anqi Sheng,^6^ it is imperative to address both acne and skin sensitivity in individuals with acne and sensitive skin.

*Guaiacum officinale* (GO), an evergreen tree native to the West Indies and northern South America,^7^, ^8^ is known for its rich resin content of lignans and polyphenols, including guaiacolol, 2‐methoxy‐phenol, and o‐hydroxyanisole, among others. This plant is traditionally used to treat inflammatory conditions such as angina, tonsillitis, and rheumatoid arthritis.^9^ Modern studies have validated its anti‐inflammatory, antioxidant, and antitumor properties, possibly attributed to the pharmacological activity of the resin.^10^ *Rhodomyrtus tomentosa* (RT), a flowering plant native to Southeast Asia, which fruits rich in flavonoids (Rhodomyrtone), phenolic acids, and various bioactive constituents, traditionally employed in Chinese medicine for treating urinary tract infections.^11^ Since 2000, researchers have demonstrated antibacterial, antimalarial, antifungal, antioxidant, and anti‐inflammatory activities of RT.^12^ Although the GO and RT extracts have been reported to have antioxidant and anti‐inflammatory activities, their synergistic effects have not been investigated in this regard, and further, their potential in the treatment of acne vulgaris is unknown.

In this study, we investigated the antioxidative and anti‐inflammatory activities of GO, RT, and their combination in vitro. The efficacy of a cream containing this combination for treating acne vulgaris and facial redness was evaluated at baseline and after 7 and 28 days of use. Self‐assessment questionnaires were collected to evaluate subjects' satisfaction after 28 days. The results showed that GO and RT extracts possess antioxidant activities, and their synergistic anti‐inflammatory efficacy was significantly stronger than individual use. The cream significantly improved facial acne and redness without causing skin irritation. This novel combination provided an alternative treatment for acne vulgaris, especially for patients with mild to moderate acne on sensitive skin.

## 2 MATERIALS AND METHODS

### 2.1 Experimental materials

The *G. officinale* extract, 10% dry matter dissolved in a water/butane‐1,2‐diol (85/15, v/v) solution, was provided by Zhenghe (Guangzhou) Biotechnology Co., Ltd. The *R. tomentosa* extract, 5% dry matter dissolved in water/propane‐1, 3‐diol (50/50, v/v), was provided by Green Tech Lab, France. Vitamin C (purity>98%) was provided by DSM. The standard products of gallic acid and rutin were procured from the National Institutes for Food and Drug Control (Beijing, China), while PGN, minimum essential medium (MEM), fetal bovine serum (FBS), and the TRIzol reagent were obtained from InvivoGen. IL‐1α, IL‐8, and TNF‐α ELISA kits, MTT kits, and dexamethasone were sourced from Neobioscience (Shenzhen, China). Additionally, a reverse transcription kit was acquired from Takara.

### 2.2 Analysis of phytochemical constituents of the GO and RT

#### 2.2.1 Total phenolic content

The total phenol content was determined using the Folin‐ Ciocalteu method with a slight modification. Absorbance was measured at 745 nm, and the concentration of gallic acid (x) and absorbance (y) were used to derive the equation of the standard curve: y = 0.1705x + 0.1373, *R* ^2^ = 0.9927. 0.5 mL of sample solutions were mixed with 0.5 mL of Folin‐Cioealteu reagent and 3.0 mL of 5% sodium carbonate solution. After incubation at 30°C for 30 min, the absorbance was measured at 745 nm. The total phenol content was expressed in milligrams of gallic acid per milliliter of sample solution, as μg GAE/mL.

#### 2.2.2 Total flavonoid content

The quantification of flavonoids was conducted using the aluminum chloride colorimetric method. 0.5 mL of sample solutions were combined with 5 mL of absolute ethanol, and 0.5 mL of 5% NaNO_2_ solution and 0.5 mL of 5% Al(NO_3_)_3_ solution were added to the mixture. After 6‐min incubation, 0.5 mL of 10% NaOH solution was introduced and allowed to culture for 30 min at room temperature. Rutin was utilized as a reference compound, and the absorbance was assessed at 500 nm. The concentration of rutin (x) and the corresponding absorbance (y) were employed to derive the equation for the standard curve: y = 0.19x–0.0468, *R* ^2^ = 0.9957. The results were expressed in terms of rutin equivalents (RE), with the unit of measurement as μg RE/mL.

### 2.3 Analysis of antioxidant activity of the GO and RT

The sample solution was diluted with ethanol to encompass weight ratios ranging from 0.01 to 100 wt%. The antioxidant activities of the extracts were determined by DPPH free radical‐scavenging assays. 2 mL of sample solutions to the test tube containing 2 mL of DPPH (0.2 mM) solution. Incubated in the dark for 30 min at room temperature and measured the absorbance at 517 nm. The positive control (PC) in this assay was Vitamin C aqueous solution at 0.01 wt%–100 wt%.

### 2.4 Analysis of anti‐inflammatory activity of the GO and RT in vitro

#### 2.4.1 Cell cultures

Human immortalized keratinocyte (HaCaT) cells were provided by the Guangzhou Customs Technology Center (Guangzhou, China) and cultured in MEM supplemented with 10% FBS and 1% penicillin–streptomycin solution. The cells were cultured for 4–6 days at 37°C under 5% CO_2_. Cells obtained after the third passage were used in this study.

#### 2.4.2 Sample preparation of cells

To assess the synergistic effects of GO and RT extracts, two extracts were mixed with volume ratios of 1:1 and 4:1 were prepared and marked as GO: RT = 1:1 and GO: RT = 4:1, respectively. The GO, RT, GO: RT = 1:1, and GO: RT = 4:1 solutions were diluted in serum‐free media to concentrations of different volume ratios (0.01%–20%, v/v).

#### 2.4.3 Cell viability

HaCaT cells (1.0 × 10^4^ cells/well) were seeded in 96‐well plates for 24 h before treatments. The different concentrations of sample solutions (0.01%–20%, v/v) were added to the cells and incubated for 24 h. Then, the culture media was removed, and 20 μL of MTT solution (5 mg/mL) were added to each well. After 4 h, the MTT solution was removed, and 100 μL of DMSO was added to each well. The absorbance was determined at 570 nm using a SpectraMax 190 microplate reader (Molecular Devices, San Jose, CA, USA).

#### 2.4.4 Real time quantitative PCR

After preincubation of HaCaT cells for 18 h, cells were exposed to different concentrations of sample solutions (0.15%, 0.30%, and 0.60%) for 30 min, and further incubated with PGN (50 μg/mL) for 24 h. DMSO was applied as the negative control (NC), and dexamethasone was applied as the PC for the test. Total RNA was extracted from each group of HaCaT cells utilizing the TRIzol technique. The cDNA was synthesized using the TaKaRa reverse transcription kit (TaKaRa, Beijing, China). The primers (Sangon, Shanghai, China) used in the study are provided in Table 1. The expression of *TLR‐2* and *TLR‐4* mRNA was normalized to that of GAPDH.

**TABLE 1:** Primer sequences used in RT‐PCR analysis.

Gene	Primer sequences
F‐TLR2	AGCACTGGCCAAAGTCTTGA
R‐TLR2	CTGTGACATTCCGACACCGA
F‐TLR4	TCCCCTGAGGCATTTAGGCA
R‐TLR4	GAAAAGGCTCCCAGGGCTAA
F‐GAPDH	AATGACCCCTTCATTGAC
F‐GAPDH	TCCACGACGTACTCAGCGC

#### 2.4.5 Quantification of IL‐1α, IL‐8 and TNF‐α by ELISA

HaCaT cells (3.0 × 10^5^ cells/well) were seeded into 12‐well plate in culture medium treated with PGN. The samples and controls were processed in accordance with the description provided in section 2.4.4. The supernatant was obtained through centrifugation at 3000 r/min and 4°C for 20 min. According to the manufacturer's instructions, the secretion of IL‐1α, IL‐8, and TNF‐α was assessed using ELISA kits (Neobioscience, Shenzhen, China), and the absorbance was measured at a wavelength of 450 nm.

### 2.5 Clinical trial

#### 2.5.1 Subjects

A total of 30 subjects, consisting of 21 females and 9 males, and aged 20–45 years, were enrolled in this study. The inclusion criteria were as follows: (a) sensitive facial skin with the lactic acid test score ≥3; (b) without any topical treatments (including medications, chemical peels, and lasers) on the face; and (c) mild to moderate acne (Global Acne Severity Scale [GEA] II to III), presenting with at least 5 comedones and papules on the face. Exclusion criteria included: (a) subjects with systemic diseases, tumors, or immunodeficiency disorders; (b) women who were pregnant or breastfeeding; (c) the presence of other skin conditions that interfered with the study, such as glucocorticoid dermatitis, rosacea, or psoriasis; (d) a documented hypersensitivity reaction to the test agent; and (e) individuals who had utilized medications for acne within the preceding month, such as glucocorticoids, antibiotics, tetracycline, etc.

The IRB number of the human clinical efficacy trial involved in this study was SLLS2023001, and all experiments were conducted in accordance with the Declaration of Helsinki. Prior to signing the informed consent form, all subjects were kept informed of all risks associated with the experiments and participated in the study voluntarily. Furthermore, the subjects have agreed to publish the pictures involved in the paper.

#### 2.5.2 Test samples and treatments

The test cream contained only 5 wt% of the GO and RT 1:1 combination as the active ingredient. The prepared test cream had shown good skin compatibility, as evidenced by the acute skin irritation test and the clinical skin patch test. All subjects applied the test cream twice daily to their faces for 28 days. During the trial period, no other products, such as cosmetics, other skincare products, or medications, were applied to the face.

#### 2.5.3 Evaluation method

The subjects were examined at baseline (D0), 7 days (D7), and 28 days (D28) in a controlled environment with a room temperature of 21 ± 1°C and a relative humidity of 50 ± 10%. Two dermatologists counted the number of facial comedones and papules. A VISIA‐CR (Canfield Scientific, Inc.) was employed to capture photographs of subjects' faces on D0, D7, and D28. Using Image Pro Plus (IPP) software to analyze the a* values of the cheek. The improvement rate of acne and redness was calculated as a percentage by the following formula: improvement rate (%) = $\;\frac{{\bar{X}}_{0}-{\bar{X}}_{1}}{{\bar{X}}_{0}}$×100%, where X_0_ represents the measured value before the used the test cream, and X_1_ denotes the measured value after the used the test cream at different time points.

#### 2.5.4 Self‐assessment of subjects

A self‐assessment questionnaire for subjects was completed at D28, which included their satisfaction and adverse reactions. Subject satisfaction of improvement in facial acne and acne leave marks was evaluated with the following 5‐point Likert scale: very satisfied‐6; satisfied‐5; slightly satisfied‐4; dissatisfied‐2; or very dissatisfied‐1. Additionally, adverse reactions to the test cream were recorded, including burning, itching, stinging, and tightness.

### 2.6 Statistical analysis

SPSS 24.0 software was used for statistical analysis, with three measurements at the same location, and the values were expressed as “mean ± SD.” One‐way ANOVA test was used for comparison between groups. The student's *t*‐test was used to calculate significance, with significance levels indicated as follows: * *p* < 0.05, **p < 0.01, and ***p < 0.001. GraphPad Prism 8.0 software was used to generate figures.

## 3 EXPERIMENTAL CONCLUSIONS

### 3.1 Analysis of phytochemical constituents and antioxidant activity of the GO and RT

To determine the phytochemical constituents present in the GO and RT extracts, we employed the Folin‐Ciocalteu method and the aluminum chloride colorimetric method to analyze the total phenolic and total flavonoid contents. The total phenol content in the GO and RT extracts were measured at 591.17 ± 4.34 μgGAE/mL and 458.98 ± 11.69 μgGAE/mL, respectively. While the total flavonoid contents were determined as 45.67 ± 3.35 μgRE/mL and 486.91 ± 10.49 μgRE/mL, respectively. Following treatment with GO and RT extracts, the scavenging efficacy of DPPH free radicals showed a dose‐dependent increase (Figure 1), with the IC_50_ values of 6.15 wt% and 0.76 wt%, respectively (Table 2). These findings suggest that phenolic and flavonoid compounds are the primary active ingredients in both the GO and RT extracts, possessing the ability to neutralize free radicals. The RT extract exhibited a more pronounced antioxidant capacity compared to GO extract.

**FIGURE 1:**
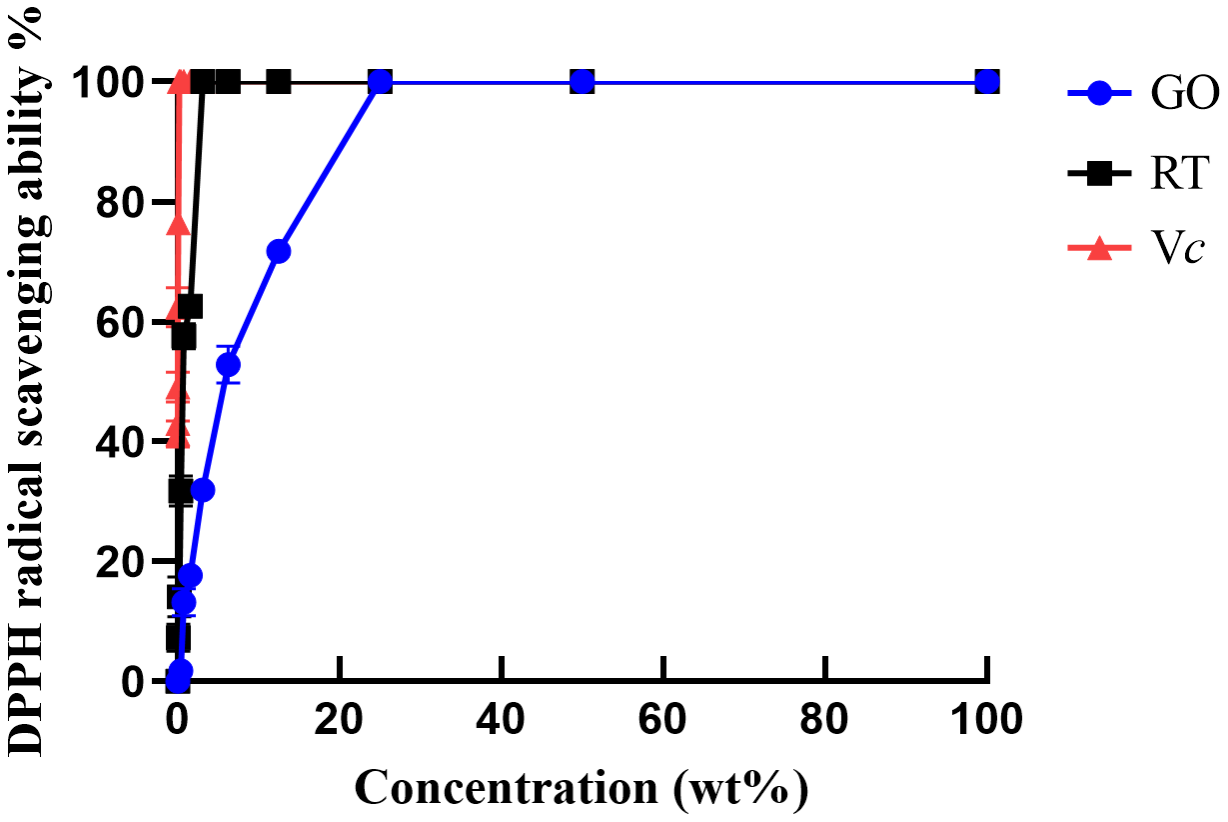
DPPH free radical scavenging activity of GO and RT extracts (0.1%–100%). Values are represented as a percentage of the control. Data are shown as mean ± standard deviation (*n* = 3).

**TABLE 2:** The content of active ingredients and DPPH radical scavenging capacity between GO, RT, and V*c.*

Samples	Total phenol (μg GAE/mL)	Total flavone (μg RE/mL)	IC_50_ of DPPH (wt%)
GO	591.17 ± 4.34	45.67 ± 3.35	6.15 ± 0.32
RT	458.98 ± 11.69	486.91 ± 10.49	0.76 ± 0.04
V*c*	—	—	0.042 ± 0.003

### 3.2 Synergistic anti‐inflammatory activity of GO and RT

#### 3.2.1 Effects of GO and RT on HaCaT cell activity

GO, RT, GO: RT = 1:1 and GO: RT = 4:1 were added to HaCaT cells to analyze their effects on cell activity. The relative cell activity of HaCaT cells remained at ≥97% in the concentration range of 0.01% to 0.60% (v/v) (Figure 2). All experiments are carried out with 0.15%, 0.30%, and 0.60% (v/v).

**FIGURE 2:**
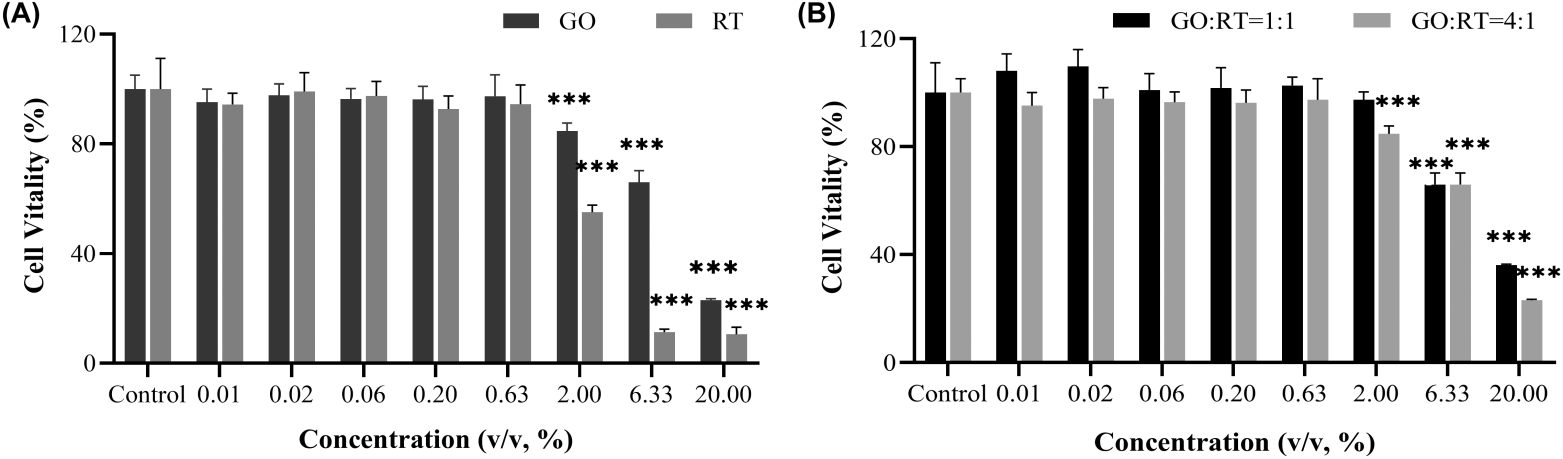
Effects of GO and RT extracts on HaCaT cell activity. (A) The combination of GO and RT had no effects on HaCaT cell activity at concentrations below 0.6% (v/v). (B) The GO: RT = 1:1 and GO: RT = 4:1 had no effect on HaCaT cell activity at concentrations less than 0.6% (v/v). Asterisks indicate a statistically significant difference between the two treatments (***p < 0.001).

#### 3.2.2 Inhibition of TLR‐2 and TLR‐4 by GO and RT

The potential effects of GO, RT, GO: RT = 1:1 and GO: RT = 4:1 on inflammation‐associated Toll‐like receptor expression were investigated by co‐treating cultured HaCaT cells with PGN and/or sample solutions. PGN significantly upregulated the expression of *TLR‐2* and *TLR‐4*. At concentrations of 0.15%, 0.30%, and 0.60%, RT, 1:1, and 4:1 samples significantly downregulated the expression of *TLR‐2* and *TLR‐4* compared to NC (Figure 3). The 1:1 sample showed a 7.93‐fold stronger inhibition of *TLR‐2* mRNA expression and a 4.38‐fold stronger inhibition of *TLR‐4* mRNA expression compared to the GO extract at 0.60%, respectively. These results suggested that GO and RT extracts may synergistically inhibit inflammation by down‐regulating the expression of toll‐like receptor‐related molecules in HaCaT cells.

**FIGURE 3:**
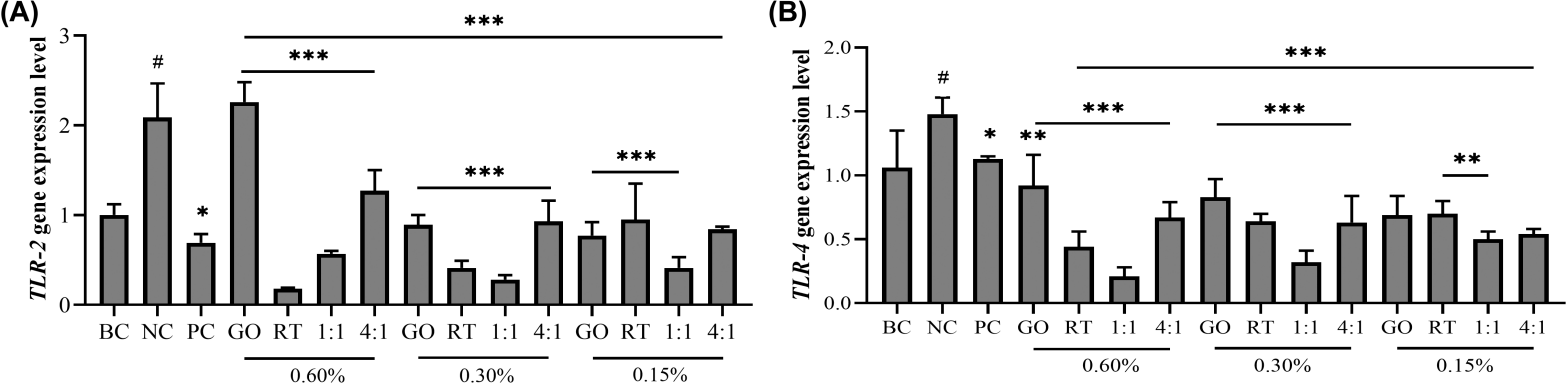
The effect of GO, RT and their combination on *TLR‐2* (A) and *TLR‐4* (B) expression. The 1:1 sample group, at 0.60% (v/v) concentration, inhibited *TLR‐2* and *TLR‐4* mRNA expression 7.93 times and 4.38 times more than that the same concentration of GO. “^#^” indicates significance for the negative control (NC) versus the blank control (BC) (^#^ *p* < 0.05). Asterisks indicate a statistically significant difference between the treatments and the NC (*p < 0.05, **p < 0.01, ***p < 0.001).

#### 3.2.3 Inhibition of pro‐inflammatory cytokine IL‐1α, IL‐8, and TNF‐α secretion by GO and RT

To investigate the inhibition of GO, RT, and their combination on the secretion of IL‐1α, IL‐8 and TNF‐α, HaCaT cells were induced by PGN with or without sample solutions. PGN significantly increased the secretion of inflammatory cytokines. At 0.15% concentration, GO inhibited the secretion of IL‐8 and TNF‐α (Figure 4). RT significantly reduced the secretion of IL‐1α and TNF‐α within 0.30%–0.60%. Notably, the 1:1 sample displayed superior anti‐inflammatory activity compared to the individual GO and RT treatments compared to NC at concentrations of 0.15%, 0.30%, and 0.60%. The 1:1 sample resulted in a 2.30‐fold stronger inhibition of IL‐8 secretion and a 2.73‐fold stronger inhibition of TNF‐α secretion compared to the equivalent concentration of GO. The combination of GO and RT extracts at a 1:1 volume ratio enhanced the anti‐inflammatory activity by reducing the secretion of inflammatory cytokines.

**FIGURE 4:**
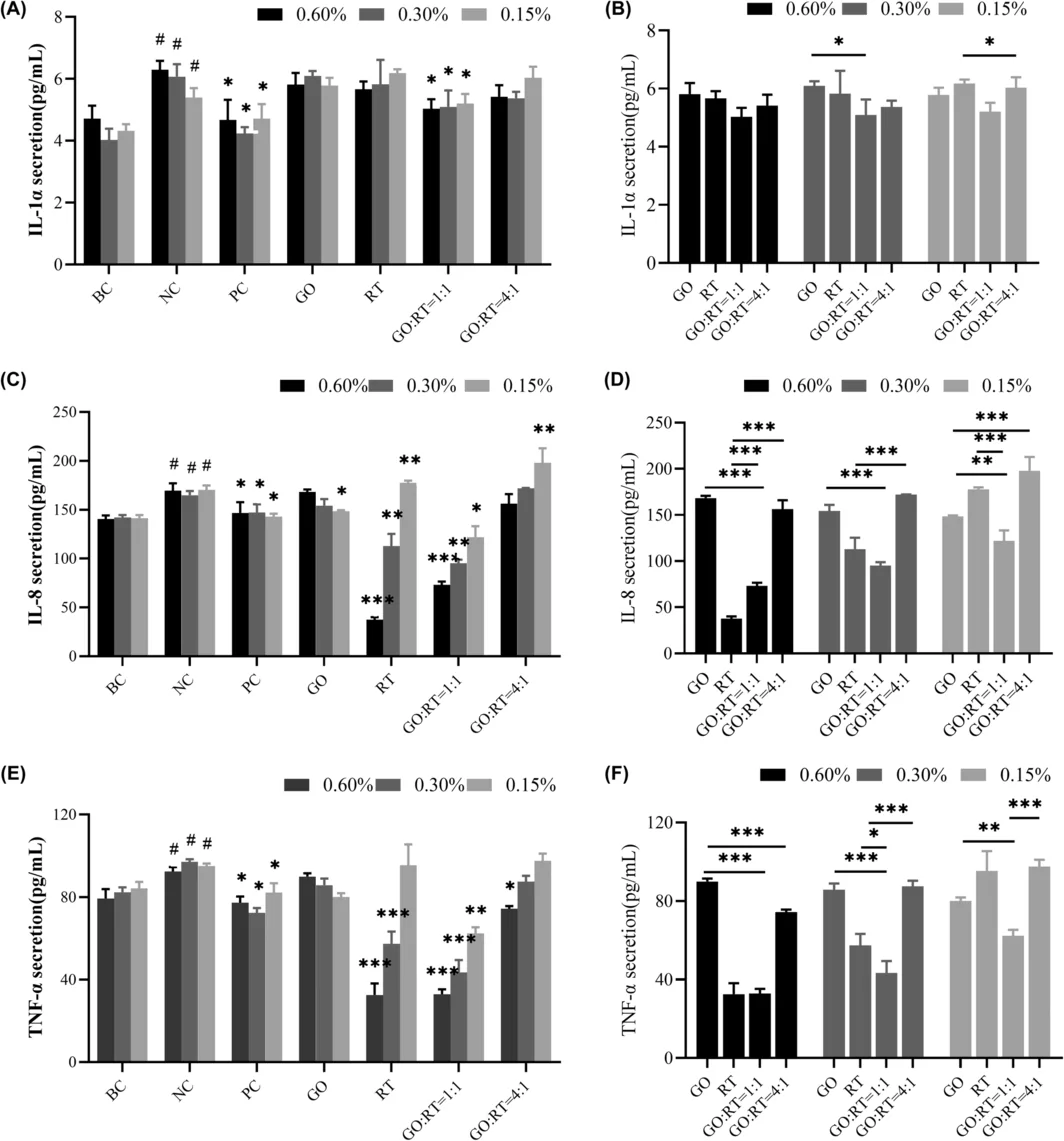
The impact of the samples on the secretion of IL‐1α (A, B), IL‐8 (C, D), and TNF‐α (E,F). The 1:1 sample group, at 0.60% (v/v) concentration inhibited TNF‐α 2.73 times more than the same concentration of GO. ^#^indicates significance for the negative control versus the blank control (BC) (^#^ *p* < 0.05). Asterisks indicate a statistically significant difference between the treatments and the NC (*p < 0.05, **p < 0.01, ***p < 0.001).

### 3.3 Clinical research

#### 3.3.1 Dermatologist assessment and instrumental evaluation

Following a 7‐day application of the test cream, the number of comedones and papules significantly decreased by 12.50% ± 5.41% and 16.67% ± 7.52% (Figure 5), respectively. After 28 days of use, the facial comedones and papules significantly decreased by 50.00% ± 2.39% and 30.65% ± 4.52%, respectively. Compared to the baseline, a significant improvement was observed in facial redness, with reductions of 23.30% ± 1.37% at D7 and 31.24% ± 0.78% at D28 (Figure 5). These findings indicated that the cream containing 5 wt% the 1:1 combination of GO and RT extracts is effective in reducing comedones and papules, as well as demonstrating comparable effectiveness in soothing and reducing skin sensitivity.

**FIGURE 5:**
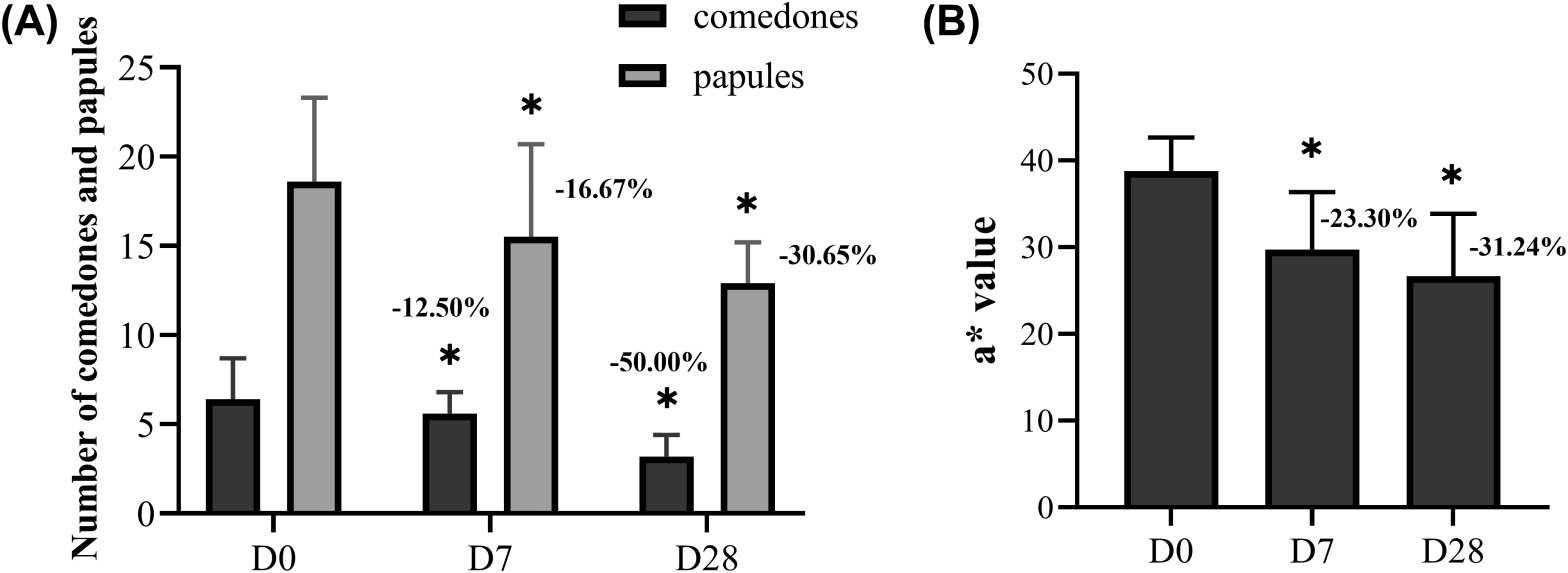
The effect of the test cream on facial acne and redness in subjects after 7 days and 28 days. (A)The 5 wt% 1:1 combination of GO and RT extracts reduced facial comedones and papules count. (B)The cream showed a significant soothing effect on facial redness of subjects. (b) Asterisks indicate a statistically significant difference between the baseline (*p < 0.05).

#### 3.3.2 Self‐assessment of subjects

The self‐assessment questionnaire, completed by the subjects after 28 days of using the test cream, indicated that all subjects were satisfied with skin barrier, the frequency and size of acne, as well as 96.67% of them were satisfied with the acne marks (Figure 7). Notably, no adverse reactions were reported.

## 4 DISCUSSION

Acne vulgaris is a prevalent dermatological condition, affecting over 650 million individuals worldwide. It is characterized by a long course of treatment, abrupt onset, complex etiology, and high propensity of recurrence, making it difficult to cure.^13^ Current therapeutic interventions for acne vulgaris include topical application of acid compounds with peeling effects such as retinoic acid, adapalene, salicylic acid, and antibacterial drugs such as erythromycin, clindamycin, and orally administered chemical synthesis antibiotics.^14^, ^15^ However, these therapeutic agents may induce adverse reactions, including skin erythema and irritation. Sensitive skin is also a common skin problem, affecting millions globally and needing immediate attention.^16^ Individuals with sensitive skin are in a prolonged state of skin barrier fragility and are unable to use the potent therapeutic agents mentioned above. The concurrent presence of facial skin sensitivity and incurable acne not only poses a double challenge in terms of physical health but also exerts a profound negative impact on the quality of life and psychological well‐being of patients. Adherence is pivotal to the successful treatment of acne vulgaris,^15^ and gentle daily skincare products clearly outperform the use of acid compounds and oral medications in this regard. Therefore, this study investigated the potential of the synergistic combination of GO and RT in a 1:1 volume ratio for treating acne vulgaris and reducing facial redness from the perspective of the care needs of patients with sensitive skin. The results of clinical trial indicated that subjects had a significant improvement in acne vulgaris severity and a* value after 28 days of using the test cream (Figures 5 and 6). The daily use of this mild and effective skincare product had no adverse effects and improved patient adherence compared to medications.

**FIGURE 6:**
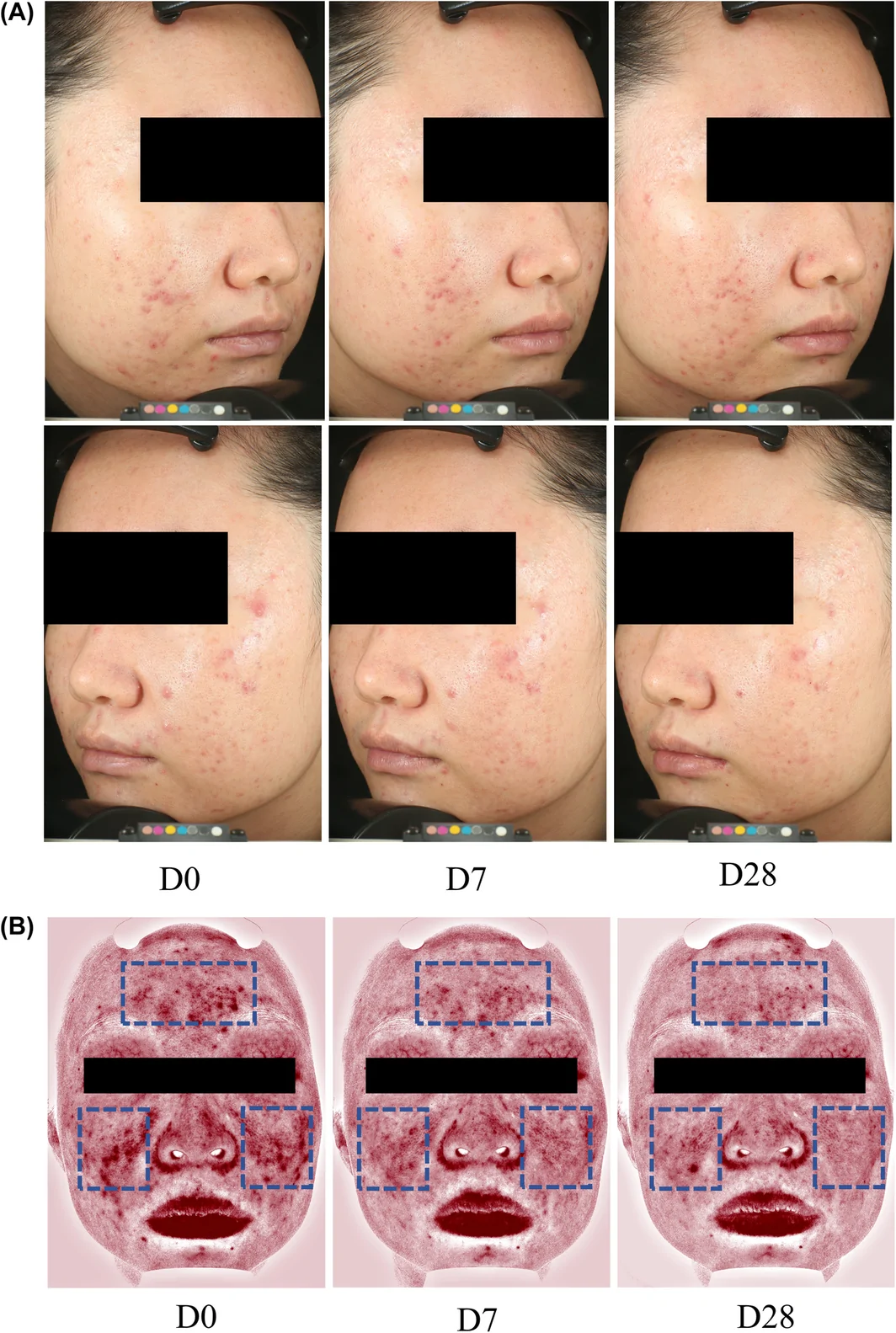
Examples of clinical improvement with the cream containing 5 wt% 1:1 combination of GO and RT extracts (A) Typical images of subjects #23 after 28 days of using the cream. (B) Typical images of subjects #30 after 28 days of using the cream.

**FIGURE 7:**
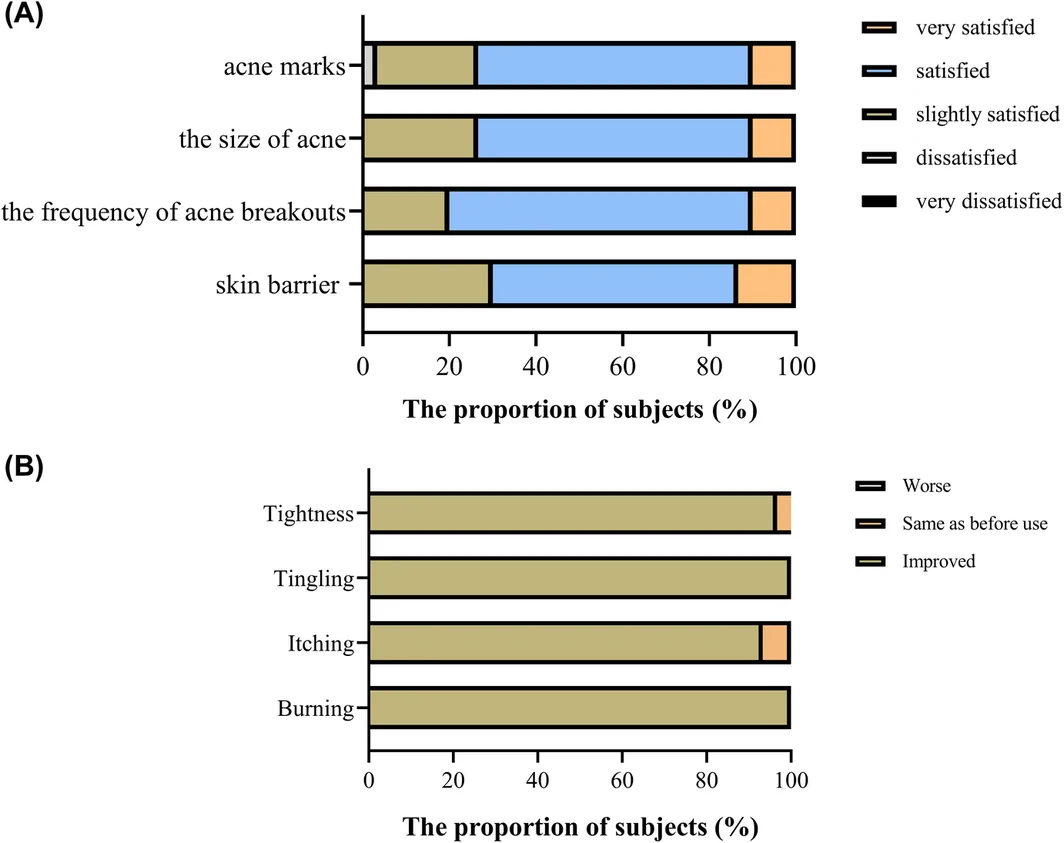
Self‐assessment questionnaire of subjects (A) All subjects were satisfied with skin barrier, the frequency and size of acne, as well as 96.67% of them were satisfied with the acne marks. (B) There were no adverse reactions after 28 days of using the cream.

Recent findings have indicated that the proinflammatory mediators induced by *C. acnes* in the sebaceous glands are closely associated with the development of acne.^17^ The main cell wall constituent of *C*. *acnes* is PGN, which is critical for cell survival and one of the best targets for bacteriostatic agents.^18^ Previous studies have demonstrated that PGN is recognized by TLRs, a class of intracellular protein molecules involved in nonspecific immunity, particularly *TLR‐2*.^18^ The *TLR‐2* mediated immune response has been identified as a pivotal factor in the inflammatory stage of acne vulgaris.^19^ Downstream signals of *TLR‐2* activation trigger the *NF‐κB* pathway, leading to the secretion of proinflammatory cytokines, chemokines, and antimicrobial compounds.^20^ Numerous researchers have used PGN to stimulate cellular production of the inflammatory cytokines IL‐1, IL‐6, and IL‐10 to predict the inhibitory effect of samples on inflammatory diseases.^21^, ^22^ After activating *TLR‐2*, PGN enhances the release of proinflammatory mediators, leading to the formation of inflammatory acne lesions. Therefore, PGN is a potent in vitro inducer for modeling acne vulgaris. In this study, we successfully established an inflammation model using PGN‐induced HaCaT cells to simulate skin acne lesions. We observed that GO and RT extracts possess anti‐inflammatory activity and inhibit the expression of *TLR‐2* and *TLR‐4* at the indicated concentrations. Furthermore, we evaluated the inhibitory effect of the 1:1 and 4:1 combination of GO and RT on PGN‐induced inflammation in vitro. Our results demonstrated that the 1:1 sample group, at 0.60% (v/v) concentration, inhibited *TLR‐2* and *TLR‐4* mRNA expression 7.93 times and 4.38 times more than that the same concentration of GO (Figure 3). Additionally, this group inhibited TNF‐α 2.73 times more than at the same concentration of GO (Figure 4), indicating the promising synergistic effects in inhibiting acne inflammation. These results provide an alternative for the development of inflammatory acne models and offer technical guidance for the application of a synergistic combination of multiple plant extracts in skincare products for the treatment of acne.

GO has traditionally been known as the “tree of life”, its therapeutic effect on pharyngitis and has been used as an anti‐inflammatory and antibacterial agent.^23^ In a recent study,^24^ after 28 days of using skincare products containing RT extracts, a redistribution of *C. acnes* phylogenetic types was observed on the facial skin of 17 subjects. This redistribution entailed a decrease in the abundance of phylogenetic IA1 type, coupled with an increase in phylogenetic II and III types. These findings suggest that RT extracts has a regulatory effect on the skin surface microbiota, aligning with the prevailing theory of skin microbiome balance. Dysbiosis of the skin microbiome, such as imbalances among its members or phylotypes, may be the one of primary cause of acne vulgaris.^25^ In this experiment, the microbiome‐regulating effect of RT extracts was synergistic with the anti‐inflammatory and antibacterial effect of GO extracts, which enhanced the inhibiting effect of acne inflammation in vitro. Based on the results of in vitro experiments, a 5 wt% of GO and RT 1:1 combination was applied into skincare products. After 28 consecutive days of use, the subjects exhibited a significant decrease in facial comedones, papules, and redness (Figures 5 and 6). This study elucidates the synergistic effects of GO and RT extracts on treating facial acne vulgaris and redness. In addition, the subjects reported no adverse effects when using the cream.

## 5 CONCLUSION

The experimental data suggest that the novel combination of GO and RT extracts demonstrates potential antioxidant capabilities and anti‐inflammatory effects in vitro. After 28 days of using the test cream containing 5 wt% of GO and RT in a 1:1 combination, subjects showed significant improvement in acne vulgaris and facial redness. This combination of active ingredients provides a balanced, multifaceted approach to acne management for individuals with sensitive skin. Nonetheless, further research is necessary to comprehensively elucidate the underlying mechanism of this synergistic combination and to provide a more scientific approach to treating acne in patients with sensitive skin.

## AUTHORS CONTRIBUTIONS

J. Z: Conception and design, acquisition of data, analysis and interpretation of data, critical revision. S. L: Conception and design, project administration, funding acquisition. W. G: Writing—review and editing, analysis, and interpretation of data. N. L: writing—original draft preparation. All authors have read and agreed to the published version of the manuscript.

## FUNDING INFORMATION

This article has no funding source.

## CONFLICT OF INTEREST STATEMENT

The authors have no conflicts of interest to declare.

## ETHICS STATEMENT

The human clinical efficacy experiments involved in this study was granted by the human research ethics committee of EviSkin Testing Technology (Guangzhou) Co., Ltd. (SLLS2023001) in February 2023, and all experiments were conducted in accordance with the Declaration of Helsinki. Prior to signing the informed consent form, all subjects were kept informed of all risks associated with the experiments and participated in the study voluntarily. Furthermore, the subjects have agreed to publish the pictures involved in the paper.

## Data Availability

The data that support the findings of this study are available from the corresponding author upon reasonable request.
